# Disruption of afferent neural circuits leads to arrhythmia in the animal model of hereditary sensory and autonomic neuropathy 6

**DOI:** 10.3389/fncir.2026.1777115

**Published:** 2026-04-08

**Authors:** Nozomu Yoshioka, Masayuki Kurose, Kazuki Tainaka, Takako Ichiki, Yousuke Tsuneoka, Hiromasa Funato, Masaki Ueno, Hayato Ohshima, Ikuo Kageyama, Hirohide Takebayashi

**Affiliations:** 1 Department of Anatomy, School of Life Dentistry at Niigata, Nippon Dental University, Niigata, Japan; 2 Division of Neurobiology and Anatomy, Graduate School of Medical and Dental Sciences, Niigata University, Niigata, Japan; 3 Division of Anatomy and Cell Biology of the Hard Tissue, Department of Tissue Regeneration and Reconstruction, Graduate School of Medical and Dental Sciences, Niigata University, Niigata, Japan; 4 Division of Functional Morphology, Department of Anatomy, Iwate Medical University, Iwate, Japan; 5 Department of Physiology, School of Dentistry, Iwate Medical University, Iwate, Japan; 6 Department of System Pathology for Neurological Disorders, Brain Research Institute, Niigata University, Niigata, Japan; 7 Division of Oral Biochemistry, Graduate School of Medical and Dental Sciences, Niigata University, Niigata, Japan; 8 Department of Anatomy, Faculty of Medicine, Toho University, Tokyo, Japan; 9 Center for Anatomical Studies, Graduate School of Medicine, Kyoto University, Kyoto, Japan

**Keywords:** arrhythmias, autonomic nervous system, dystonia musculorum mouse, Dystonin, hereditary sensory and autonomic neuropathies, sensory nervous system

## Abstract

Hereditary sensory and autonomic neuropathies (HSANs) are a group of recessive genetic disorders affecting the sensory and autonomic components of the peripheral nervous system (PNS). Compared with somatosensory dysfunctions, the pathogenesis of visceral dysfunction in HSANs remains understudied. This study investigated the neural circuit mechanisms underlying the arrhythmias observed in conditional Dystonin (*Dst*) gene-trap mice, an animal model of HSAN type VI (HSAN-VI) in which Cre recombinase inactivates *Dst* expression in selective neural circuits. Inactivation of the *Dst* gene in PNS neurons using *Advillin-Cre* caused the degeneration of sensory and sympathetic ganglionic neurons. This was accompanied by arrhythmia, characterized by increased heart rate variability and irregular pulse frequency, which was prominent under isoflurane anesthesia and occurred in the absence of protein aggregate cardiomyopathy. Furthermore, selective inactivation of the *Dst* gene in PNS sensory neurons using *Vglut2-Cre* resulted in similar dysregulation of cardiac rhythm. These findings suggest that arrhythmias caused by *Dst* mutations arise from the disruption of visceral afferent circuits, and that these neural circuits could be potential therapeutic targets for visceral dysfunction in HSAN-VI.

## 1 Introduction

Hereditary sensory and autonomic neuropathies (HSANs) comprise a group of clinically and genetically heterogeneous neurodegenerative disorders that affect the peripheral nervous system (PNS) (Rotthier et al., 2012). Patients with HSANs exhibit neurodegeneration in both the sensory and autonomic nervous systems. Because HSANs disrupt a wide range of neural networks, identifying the specific circuits responsible for individual symptoms and those amenable to targeted therapeutic intervention remains a major challenge. HSAN type VI (HSAN-VI) is caused by loss-of-function mutations in the Dystonin (*DST*) gene (Edvardson et al., 2012). In mice, naturally occurring *dystonia musculorum* (*dt*) mutation results in sensory neuron degeneration and abnormal motor behaviors, including ataxia and dystonia (Duchen et al., 1964; Horie et al., 2016). Since the identification of *Dst* as the causative gene for the *dt* phenotype (Brown et al., 1995; Guo et al., 1995), *dt* mice have been widely used to investigate the pathogenic mechanisms of HSAN-VI and to explore potential therapeutic strategies (Ferrier et al., 2014). To dissect the role of *Dst* in specific neural circuits, we previously generated a multipurpose *Dst* allele that enables cell type- or neural circuit-selective inactivation and restoration via Flip-excision (FLEX) technology (Schnütgen et al., 2005; Horie et al., 2014; Hossain et al., 2018). Using this genetic tool, we demonstrated that degeneration of proprioceptive neurons in the dorsal root ganglia (DRG) is causative for the movement disorders observed in *dt* mice (Horie et al., 2020; Yoshioka et al., 2024).

In addition to somatosensory dysfunction, patients with HSAN-VI exhibit various visceral abnormalities, including impaired cardiovascular reflexes, sexual dysfunction, pupillary abnormalities, and gastrointestinal dysmotility (Manganelli et al., 2017). However, the underlying neural circuit mechanisms remain poorly understood. Reduced gastrointestinal motility in *dt* mice has been suggested to be associated with degeneration of the vagus nerve (Lynch-Godrei et al., 2020). The vagus nerve comprises parasympathetic efferent fibers as well as visceral afferent fibers (Waise et al., 2018). Notably, signs of neurodegeneration have been reported in the vagal ganglia, which include visceral sensory neurons, in *dt* mice (Ichikawa et al., 2006; Lynch-Godrei et al., 2020). These observations raise the possibility that disruption of visceral afferent circuits contributes to visceral dysfunctions in HSAN-VI.

The *Dst* gene encodes tissue-specific isoforms, including DST-a, DST-b, and DST-e, which are predominantly expressed in neural, muscular, and cutaneous tissues, respectively (Künzli et al., 2016; Horie et al., 2017; Yoshioka, 2024). These major DST isoforms are thought to play essential roles in maintaining the integrity of their respective tissues by acting as cytoskeletal linker proteins. We previously reported that isoform-specific *Dst-b* mutant mice develop late-onset protein aggregate-associated cardiomyopathy and arrhythmia, suggesting that cell-autonomous defects in cardiomyocytes can lead to dysregulation of cardiac function (Yoshioka et al., 2022). This observation was subsequently supported by a clinical report identifying *DST-b*-specific mutations as a cause of congenital myopathy and cardiomyopathy in human patients (Jacob et al., 2025). Nevertheless, it remains unclear whether the cardiovascular dysregulation observed in patients with HSAN-VI arises primarily from abnormalities in neural circuits or from intrinsic defects in cardiomyocytes. To address this issue, we performed conditional inactivation of the *Dst* gene in sensory and/or autonomic nervous systems to elucidate the respective contributions of PNS neural circuits to cardiac rhythm regulation in HSAN-VI.

## 2 Materials and methods

### 2.1 Animals

We used *Dst*^*Gt*(*E182H05*)^ mice (*Dst^Gt^*; MGI: 3917429, Horie et al., 2014) derived from the ES clone obtained from GGTC (ID: E182H05), and *Dst-b^E2610Ter^* mice (*Dst^em1Htak^*, MGI:7423674, Yoshioka et al., 2022). *Dst^Gt^* mice were crossed with several Cre-driver mice including PNS neuron-selective *Advillin* (*Avil*)*-Cre* mice (*Avil^tm2(cre)Fawa^*; MGI:4459942, Hasegawa et al., 2007; Zhou et al., 2010), and glutaminergic neuron-selective *Vglut2-Cre* mice (*Slc17a6^tm2(cre)Lowl^*/J; MGI: 5300532, Vong et al., 2011). *Ai14* reporter mice (B6. Cg-*Gt(ROSA)26Sor^tm14(CAG-tdTomato)Hze^*/J; MGI:3813512, Madisen et al., 2010) were crossed with *Avil-Cre* mice or *Vglut2-Cre* mice. The functional *Dst^Gt-inv^* allele was inverted from *Dst^Gt^* allele by FLP recombinase, and mutant *Dst^Gt-DO^* allele was inverted from *Dst^Gt-inv^* allele by Cre recombinase (Horie et al., 2014). Mutant mice of *Dst^Gt^*, *Dst^Gt-inv^*, and *Dst^Gt-DO^* were maintained in each line. To perform Cre-mediated conditional inactivation of the *Dst* expression, gene-trap mice harboring multipurpose *Dst* alleles were crossed with each Cre driver mouse. The animal experiments were approved by the Internal Review Board of Niigata University. Mice were maintained at 23 ± 3 °C, 50 ± 10% humidity, and 12 h light/dark cycle with food and water available *ad libitum*.

### 2.2 Tissue preparations and *in situ* hybridization

*In situ* hybridization was carried out as described in previous studies (Yoshioka et al., 2020, 2022, 2024). For tissue preparation, mice were euthanized by intraperitoneal injection of pentobarbital sodium (100 mg/kg body weight) and transcardially perfused with 0.01 M phosphate-buffered saline (PBS), followed by ice-cold 4% paraformaldehyde (PFA) in 0.1 M phosphate buffer (PB), pH 7.4. Dissected tissues were immersed in the same fixative overnight. Neural tissues were cryoprotected by immersion in 20% sucrose in 20 mM PBS (pH 7.4) until they sank, frozen in liquid nitrogen, embedded in Tissue-Tek OCT compound (Sakura Finetek Japan, Tokyo, Japan), and sectioned at 16 μm using a cryostat (Leica CM1850 UV; Leica, Wetzlar, Germany; HM525 NX; Thermo Fisher Scientific, Waltham, MA). Heart tissues were dehydrated through a graded ethanol series followed by xylene and embedded in paraffin (Paraplast Plus^®^, P3683; Sigma-Aldrich, St. Louis, MO, USA). Consecutive 10-μm-thick paraffin sections were cut using a rotary microtome (HM325; Thermo Fisher Scientific), mounted on MAS-coated glass slides (Matsunami Glass, Osaka, Japan), and air-dried overnight on a hot plate at 37 °C. For *In situ* hybridization the following probes were used: mouse *Dst-plakin* (Genbank accession number, NM_001276764, nt 2185–3396), and mouse *Nppa*, also known as atrial natriuretic peptide (*ANP*, GenBank accession, BC089615, nt 124–529).

### 2.3 Immunohistochemistry

Immunohistochemical analyses were performed on paraffin sections as described in previous studies (Yoshioka et al., 2020, 2022, 2024). Deparaffinized sections were subjected to microwave irradiation in 10 mM citric acid buffer, pH 6.0 for 5 min, or frozen sections were used without antigen retrieval. Sections were incubated overnight at 4 °C with following primary antibodies: rabbit polyclonal anti-PGP9.5 antibody (1:1000; ENZO Life Sciences, Farmingdale; ADI-905-520-1), rabbit polyclonal anti-TH antibody (1;500; Pel-Freez Biologicals, Rogers, AR; P40101), rabbit polyclonal anti-ATF3 antibody (1:2000; Santa Cruz Biotechnology, Dallas, TX; sc-188), rabbit polyclonal anti-Sprr1a antibody (1:1000; ABclonal Technology, Woburn, MA; A17535), rabbit polyclonal anti- HspB1/HSP25 antibody (1:800; ABclonal Technology; A11156), rabbit polyclonal anti-HSPA1L antibody (1:500; ABclonal Technology; A1856), rabbit polyclonal anti-VAchT antibody (1:500; GeneTex, Irvine, CA; GTX133251), and mouse monoclonal anti-p62 antibody (1:200; BioLegend, San Diego, CA; MMS-5034) diluted in 0.1 M PBS with 0.01% triton X-100 (PBST) containing 0.5% skim milk. Sections were then incubated with horseradish peroxidase-conjugated secondary antibody (1:200; MBL, Nagoya, Japan) diluted in PBST containing 0.5% skim milk for 60 min at 37 °C. Between each step, sections were rinsed in PBST for 15 min. After rinsing sections in PBST, immunoreaction was visualized in 50 mM Tris buffer (pH 7.4) containing 0.01% diaminobenzidine tetrahydrochloride and 0.01% hydrogen peroxide at 37 °C for 5 min. Sections were dehydrated through ethanol-xylene and coverslipped with Bioleit (23–1002; Okenshoji, Tokyo, Japan). For immunofluorescent staining, sections were incubated in mixtures of Alexa488- or Alexa594-conjugated antibodies (1:200; Invitrogen, CA) for 60 min at 37 °C. Mounted sections were air-dried and coverslipped. Digital images were taken with a microscope (BX53; Olympus, Tokyo, Japan) equipped with a digital camera (DP74, Olympus) and a confocal laser scanning microscopy (FV-1200, Olympus). TIF files were processed with Photoshop software (Adobe, San Jose, USA).

### 2.4 Measurements of electrocardiogram signal and quantification

Electrocardiogram (ECG) was recorded as described in previous study under anesthesia (Yoshioka et al., 2022). Mice were exposed to 3.5% isoflurane (Pfizer Inc., NY) for induction of anesthesia and then switched to 1.5% isoflurane for maintenance, during which ECG was recorded for a duration of 5 min. Three needle electrodes were inserted into the right and left forelimbs for recording, and right hindlimb for grounding. The ECG signals were amplified using AC amplifier (band pass: 0.1–1 kHz), and the signals were digitized with A/D converter (Power 1401, Cambridge Electronic Design Ltd., Cambridge, UK), at sampling rate of 2 kHz. The recorded ECG signals were analyzed using LabChart Pro software (version 8.1.30; ADInstruments, Bella Vista, Australia) with the ECG Analysis Module. Segments containing significant noise or motion artifacts were identified by visual inspection and excluded from the analysis to ensure data quality. Three epochs were defined for analysis: (i) the induction period under 3.5% isoflurane, (ii) the immediate post-transition period to 1.5% isoflurane, and (iii) the stabilized maintenance period 5 min post-transition. Within each epoch, the heart rate (HR), RR interval, and the standard deviation (SD) of RR intervals were calculated as indices of heart rate variability (HRV). To analyze ECG waveforms, at least 30 consecutive beats were averaged to determine the following parameters: the PR (PQ) interval (from the onset of the P wave to the start of the QRS complex), the QRS duration (from the start of the Q wave to the end of the S wave), and the QT interval (from the beginning of the QRS complex to the end of the T wave).

### 2.5 ECG recording in freely moving mice

To evaluate cardiac activity in unanesthetized mice, we performed chronic ECG recordings using a custom-built system in freely moving mice. Mice were anesthetized with 3.5% isoflurane. Following a midline skin incision, enamel-coated copper wires were implanted and positioned to sandwich the heart. This configuration was chosen to ensure optimal signal detection of the cardiac vector, thereby maximizing the R-wave amplitude and signal-to-noise ratio. The electrode leads were routed subcutaneously to the dorsal region and exteriorized. A stainless steel screw electrode was implanted into the occipital bone to serve as the system ground. Subsequently, all leads were soldered to a custom-made head-mounted connector. To ensure long-term stability, the connector was rigidly secured to the bone using dental resin (Super-Bond; Sun Medical, Shiga, Japan). Finally, the skin incisions were carefully sutured to facilitate post-operative recovery. Following a recovery period, ECG signals were recorded from free-moving mice. To initiate recording, the head-mounted connector was linked to the data acquisition system using a custom-made recording system (Unique Medical, Tokyo, Japan). This system, which extended from the head-mounted connector through an electrical swivel (slip-ring) to the amplifier, allowed for unrestricted movement within the observation cage. This flexible setup enabled stable, long-term data acquisition in a freely moving state without movement-induced stress. The ECG signals were amplified using AC amplifier (band- pass filter: 0.1–1 kHz), and the signals were digitized with A/D converter (Power 1401, Cambridge Electronic Design Ltd.), at sampling rate of 2 kHz. The recorded ECG signals were analyzed using LabChart Pro software (version 8.1.30; ADInstruments) with the ECG Analysis Module. Following the connection of the recording cable to head-connector, mice were allowed to habituate for 30 min before data collection started. For analysis, a 1-min segment was extracted during periods characterized by a stable baseline and minimal waveform variability. Segments containing significant noise or motion artifacts were identified by visual inspection and excluded from the analysis. To analyze ECG waveforms, at least 30 consecutive beats were averaged.

### 2.6 Tissue clearing protocol and imaging

The brain and spinal cord were made transparent for imaging using clear unobstructed brain/body imaging cocktails and computational analysis (CUBIC) protocol (Tainaka et al., 2018), as described previously (Yoshioka et al., 2024). The brains and spinal cords were dissected from mice following perfusion with PBS, pH 7.4, and 4% PFA in 0.1 M PB. The tissues were postfixed overnight in same fixative at 4 °C and washed with PBS. The samples were immersed in CUBIC-L [10% polyethylene glycol mono-p-isooctyphenyl ether (12969–25, Nacalai Tesque, Kyoto) and 10% N-butyldiethanolamine (B0725, Tokyo Chemical Industry, Tokyo) in water] with shake at 37 °C for 5 days. CUBIC-L was exchanged at 2 days after the immersion. The samples were then washed three times with PBS at room temperature for 2 h and then immersed in 50% CUBIC-R [45% 2,3-dimethyl-1-phenyl-5-pyrazolone (D1876, Tokyo Chemical Industry) and 30% nicotinamide (N0078, Tokyo Chemical Industry), pH adjusted to approximately 8 to 9 with N-butyldiethanolamine (B0725, Tokyo Chemical Industry, Tokyo) in water] diluted in water at room temperature for 5 h and then gently shaken in CUBIC-R at room temperature overnight. The samples were immersed in new CUBIC-R and kept until the microscopic observation. 3D fluorescent images were acquired with light sheet fluorescence microscopes (MVX10-LS, Olympus).

### 2.7 Statistical analysis

Statistical analysis was performed using *t*-test (Control vs. cGT) or two-way repeated measures analysis of variance (ANOVA) with genotype (Control vs. cGT) and epoch (i, ii, and iii) as the main factors. Post-hoc comparisons were conducted by Tukey to identify specific differences between groups. These analyses were carried out using the Easy R (EZR, Saitama Medical Center, Jichi Medical University, Japan, Kanda, 2013). Sample size (n) is the number of animals in each genotype.

## 3 Results

### 3.1 PNS neuron-selective inactivation of *Dst* gene leads to neurodegeneration

**Figure 1:**
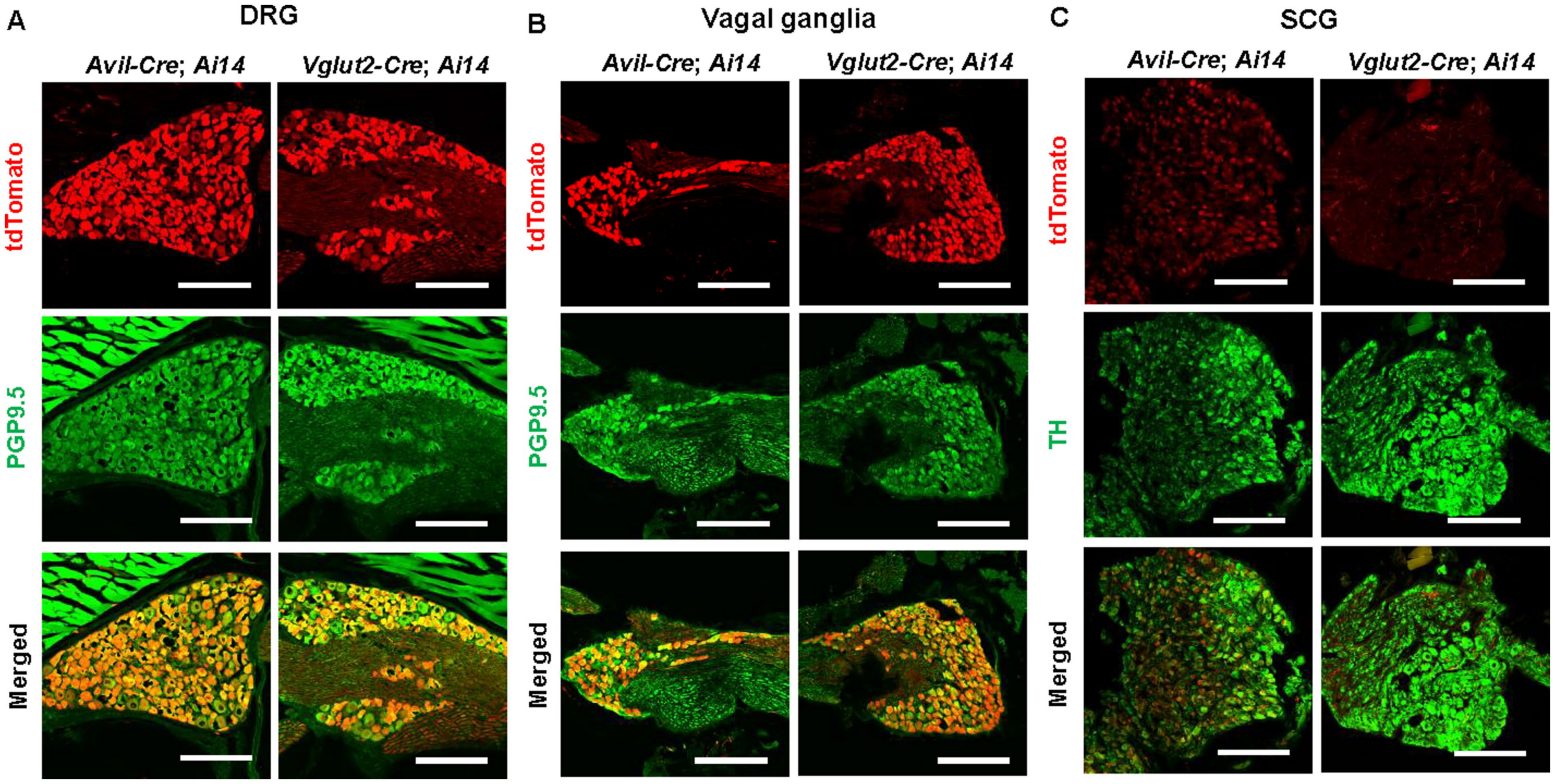
Cre-mediated reporter expression in sensory and autonomic ganglia of *Advillin-Cre*; *Ai14* and *Vglut2 − Cre*; *Ai14* mice. **(A–C)** The distribution of tdTomato fluorescent (red) was examined in the dorsal root ganglia (DRG), vagal ganglia, and superior cervical ganglia (SCG) of *Advillin* (*Avil*)*-Cre*; *Ai14* and *Vglut2-Cre*; *Ai14* mice to compare Cre-recombinase activity driven by the *Avil* and *Vglut2* promoters. The *Ai14* reporter allele expresses tdTomato following Cre-mediated recombination. **(A)** In the DRG of both *Avil-Cre*; *Ai14* and *Vglut2-Cre*; *Ai14* mice, tdTomato is expressed by many sensory neurons positive for PGP9.5. **(B)** In the vagal ganglia of *Avil-Cre*; *Ai14* and *Vglut2*-*Cre*; *Ai14* mice, tdTomato is expressed by PGP9.5-positive sensory neurons. **(C)** In the SCG of *Avil-Cre*; *Ai14* mice, tdTomato is expressed in nearly all sympathetic ganglionic neurons positive for tyrosine hydroxylase (TH). In contrast, in the SCG of *Vglut2*-*Cre*; *Ai14* mice, TH-positive ganglionic neurons lack tdTomato expression, although some tdTomato-positive fibers are observed within the SCG. Scale bars, 150 μm.

Previously, we generated multipurpose gene-trap mice carrying the *Dst^Gt^* allele, which enables both Cre-mediated inactivation and restoration of *Dst* (Horie et al., 2014; Yoshioka et al., 2024). To achieve inactivation of *Dst* gene expression in PNS neurons, we used *Avil-Cre* mice, in which Cre-mediated recombination occurs in sensory neurons and sympathetic ganglionic neurons (Zurborg et al., 2011; Hunter et al., 2018). To confirm PNS neuron-selective recombination, *Avil-Cre* mice were crossed with *Ai14* reporter mice, in which tdTomato expression is induced by Cre-mediated recombination (Figure 1). In *Avil-Cre*; *Ai14* mice, tdTomato was observed in sensory neurons of the dorsal root ganglia (DRG) (Figure 1A) and vagal ganglia (Figure 1B), as well as in sympathetic ganglionic neurons of the superior cervical ganglion (SCG) (Figure 1C). These results indicate efficient Cre-mediated recombination in both PNS afferent and efferent neurons by the *Avil-Cre* mice.

**Figure 2:**
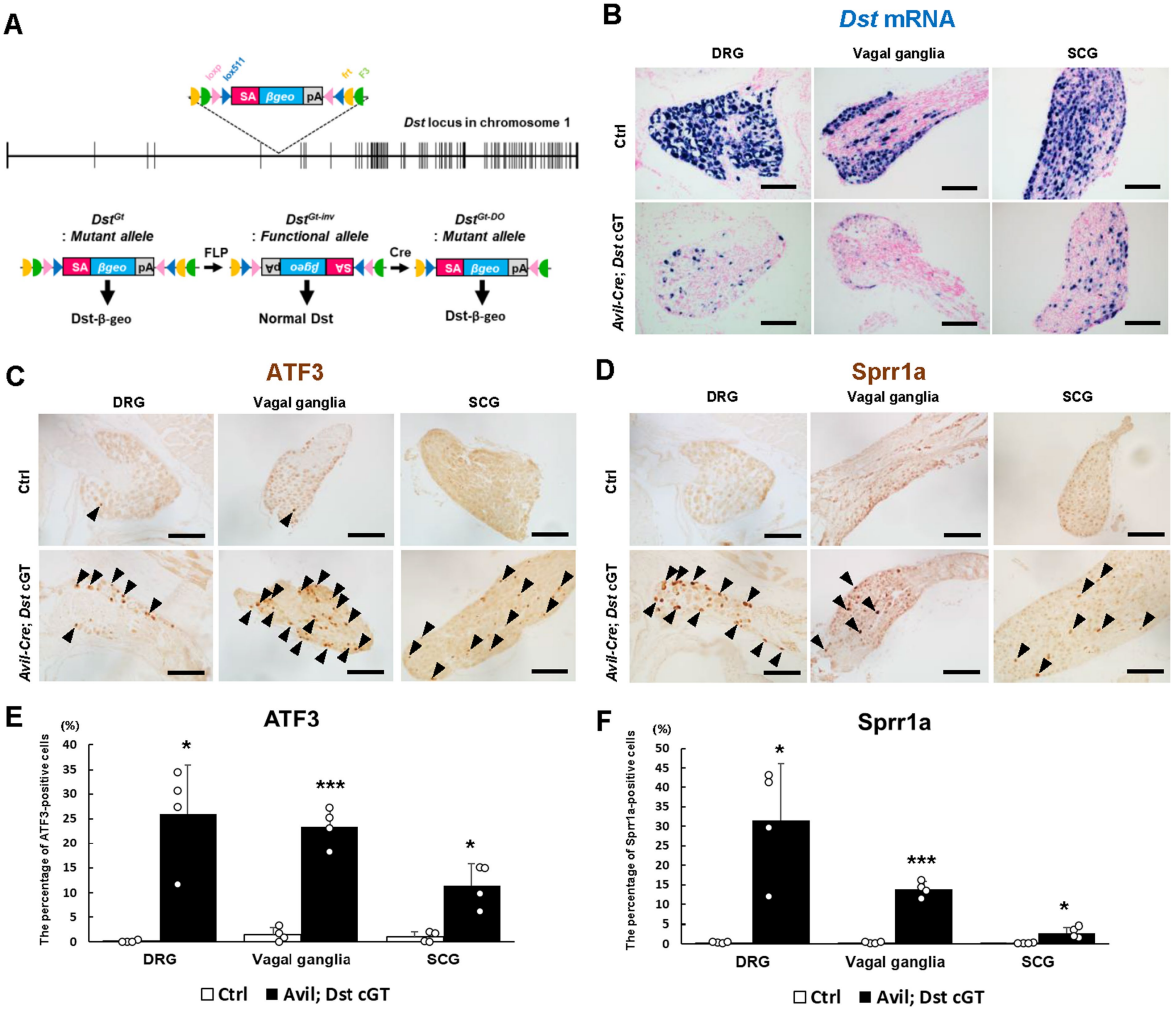
Histological analyses of sensory and autonomic ganglia in *Avil-Cre*; *Ai14* mice. **(A)** A schematic representation of the *Dst* locus containing conditional gene-trap cassette. The gene-trap cassette consists of a splice acceptor (SA) sequence, the reporter gene *βgeo*, and polyadenylation (pA) termination signal. Pairs of inversely oriented target sites for Cre recombinase (*loxP* and *lox5171*: triangles) and FLP recombinase (*Frt* and *F3*: half circles) flank the gene-trap cassette. FLP- or Cre-mediated recombination irreversibly converts the mutant *Dst^Gt^* allele into the functional *Dst^Gt-inv^* allele, and the functional *Dst^Gt-inv^* allele into the mutant *Dst^Gt-DO^* allele. The *Dst^Gt^* and *Dst^Gt-DO^* alleles express nonfunctional Dst-β-geo, whereas the *Dst^Gt-inv^* allele expresses functional Dst. **(B)** *In situ* hybridization analyses performed at 4 weeks of age. In *Avil-Cre*; *Dst* cGT mice, *Dst* mRNA levels are reduced in sensory neurons of the DRG and vagal ganglia, as well as in sympathetic ganglionic neurons of the SCG. **(C,D)** Neurodegenerative changes in the DRG, vagal ganglia, and SCG of *Avil-Cre*; *Dst* cGT mice at 4 weeks of age. Expression of the neuronal injury markers ATF3 **(C)** and Sprr1a **(D)** is increased in the DRG, vagal ganglia, and SCG of *Avil-Cre*; *Dst* cGT mice (arrowheads). Scale bars, 100 μm. (**E,F**) Quantitative analysis of the percentage of ATF3-positive **(E)** and Sprr1a-positive cells **(F)** in DRG, vagal ganglia, and SCG of Ctrl mice and *Avil-Cre*; *Dst* cGT mice (*n* = 4 Ctrl mice; *n* = 4 *Avil-Cre*; *Dst* cGT mice). * and *** denotes statistically significant difference at *p* < 0.05 and *p* < 0.005.

The *Dst^Gt^* allele carries a multipurpose gene-trap cassette that enables both Cre-mediated inactivation and restoration of *Dst* through the inversion of the cassette. The mutant *Dst^Gt-DO^* allele was inverted from functional *Dst^Gt-inv^* allele by Cre recombinase (Figure 2A). For conditional gene-trap (cGT) experiments, male *Avil-Cre*; *Dst^Gt-DO/WT^* mice were crossed with female *Dst*^*Gt-inv*/*Gt-inv*^ mice (Supplementary Figure 1). The *Avil-Cre*; *Dst* cGT mice (*Avil-Cre*; *Dst*^*Gt-DO*/*Gt-inv*^) were obtained as one-fourth of the offspring, and mice of the other genotypes were used as littermate controls (Ctrl). To validate PNS neuron-selective inactivation of *Dst* expression, *Dst* transcripts were visualized by *in situ* hybridization (Figure 2B). In Ctrl mice, *Dst* mRNA was detected in sensory neurons of the DRG and vagal ganglia, as well as in sympathetic ganglionic neurons of the SCG. In contrast, *Dst* mRNA levels were markedly reduced in these neurons in *Avil-Cre*; *Dst* cGT mice.

Sensory neurodegeneration is a pathological hallmark of *dt* mice (Duchen et al., 1964; Horie et al., 2016). We assessed neurodegenerative signs by analyzing the expression of ATF3 and Sprr1a in *Avil-Cre*; *Dst* cGT mice at 1 month of age (Figures 2C,D). ATF3 is a marker of neural injury due to its selective induction following nerve injury (Takeda et al., 2000; Tsujino et al., 2000; Kiryu-Seo et al., 2016). We previously reported that ATF3 expression is induced in DRG sensory neurons of *Dst* mutants (Yoshioka et al., 2024). Consistently, in *Avil-Cre*; *Dst* cGT mice, ATF3 expression was markedly induced in neurons of the DRG, vagal ganglia, and SCG, whereas it was scarcely detected in Ctrl mice (Figure 2C). Sprr1a is upregulated in DRG sensory neurons and spinal motor neurons following axotomy (Bonilla et al., 2002). We found that Sprr1a was also increased in sensory neurons of the DRG and vagal ganglia, as well as in sympathetic ganglionic neurons of the SCG in *Avil-Cre*; *Dst* cGT mice (Figure 2D). The expression of ATF3 and Sprr1a was quantified in DRG, vagal ganglia, and SCG of Ctrl and *Avil-Cre*; *Dst* cGT mice (Figures 2E,F). In all ganglia, the expression of both ATF3 and Sprr1a was significantly increased. However, the upregulation tended to be lower in the SCG compared with DRG and vagal ganglia. The expression of ATF3 and Sprr1a was also examined in parasympathetic ganglionic neurons of cardiac ganglia. These neural injury markers were scarcely induced in the systemic *Dst*-knockout (*Dst* GT) mice (Supplementary Figure 2) and *Avil-Cre*; *Dst* cGT mice (Supplementary Figure 3). Collectively, these histological analyses indicate that both PNS sensory (afferent) and sympathetic ganglionic (efferent) neurons of *Avil-Cre*; *Dst* cGT mice exhibit signs of neurodegeneration, while cardiac ganglionic neurons seem to be unaffected.

### 3.2 PNS neuron-selective inactivation of *Dst* gene causes dysregulated heart rate

**Figure 3:**
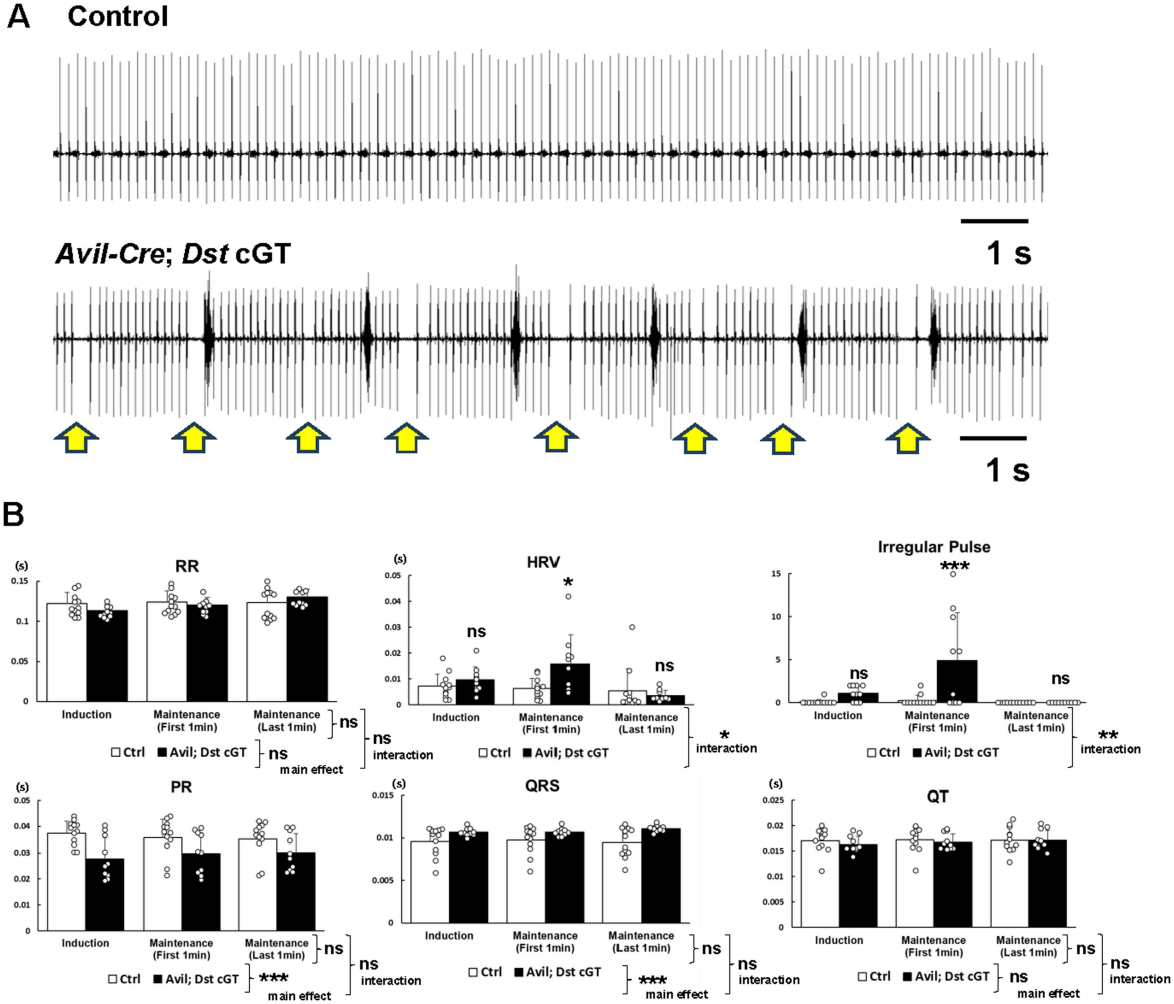
Electrocardiogram recordings from *Avil-Cre*; *Dst* cGT mice under anesthesia. **(A)** Representative electrocardiogram (ECG) traces from Ctrl and *Avil-Cre*; *Dst* cGT mice under anesthesia. Arrows indicate abnormal skipping of P waves. **(B)** Quantification of mean RR intervals, HRV, frequency of irregular pulse, PR interval, QRS duration, and QT interval (*n* = 12 Ctrl mice; *n* = 10 *Avil-Cre*; *Dst* cGT mice, at 9–12 months of age). ECG was quantified in three epochs: (i) 1-min of the induction anesthesia period, (ii) first 1-min of maintenance anesthesia period, and (iii) last 1-min of the 5-min maintenance period. *, **, and *** denotes statistically significant difference at *p* < 0.05, *p* < 0.01, and *p* < 0.001 and ns means not statistically significant (*p* > 0.05), using two-way ANOVA. Data are presented as mean ± SD.

Previously, we reported arrhythmias in isoform-specific *Dst-b* mutant mice, in which *Dst* expression is disrupted in cardiomyocytes (Yoshioka et al., 2022). These *Dst-b* mutants exhibited change in the ECG waveform, characterized by QT prolongation and premature contraction. In the present study, ECG recordings were obtained under isoflurane anesthesia in *Avil-Cre*; *Dst* cGT and Ctrl mice at 9–12 months of age (Supplementary Figure 4). ECG data were compared across three distinct epochs: induction, and the first and last minutes of a 5-min maintenance period. In the first minute of maintenance anesthesia, *Avil-Cre*; *Dst* cGT mice tended to exhibit highly variable heart rate rhythms compared with Ctrl mice (Figure 3A). HRV was quantified by the SD of RR intervals. The HRV of *Avil-Cre*; *Dst* cGT mice was significantly higher than that of Ctrl mice (genotype effect, *F* (1, 60) = 4.20, *p* < 0.05; epoch effect, *F* (2, 60) = 5.47, *p* < 0.01; genotype × epoch interaction, *F* (2, 60) = 4.12, *p* < 0.05, two-way ANOVA) (Figure 3B). The frequency of irregular pulse, defined as the number of instances exceeding ±3 × SD of RR intervals, was also significantly increased in *Avil-Cre*; *Dst* cGT mice compared with Ctrl mice (genotype effect, *F* (1, 60) = 12.04, *p* < 0.001; epoch effect, *F* (2, 60) = 8.18, *p* < 0.001; genotype × epoch interaction, *F* (2, 60) = 6.72, *p* < 0.01, two-way ANOVA) (Figure 3B). Although significant differences in HRV and irregular pulse were observed during immediate post-transition period, these effects diminished by the stabilized maintenance period. Two-way ANOVA revealed that *Avil-Cre*; *Dst* cGT mice exhibit a significantly widened QRS complex (genotype effect, *F* (1, 60) = 14.63, *p* < 0.001; epoch effect, *F* (2, 60) = 0.07, *p* > 0.05; genotype × epoch interaction, *F* (2, 60) = 0.48, *p* > 0.05, two-way ANOVA) and a shortened PR interval (genotype effect, *F* (1, 60) = 16.38, *p* < 0.001; epoch effect, *F* (2, 60) = 0.00, *p* > 0.05; genotype × epoch interaction, *F* (2, 60) = 0.68, *p* > 0.05, two-way ANOVA) compared with Ctrl mice. However, no significant main effect of epoch or genotype × epoch interaction for these parameters was observed. In contrast, no significant differences were observed in RR and QT intervals with respect to either genotype or epoch, and no interaction was detected (Figure 3B). Collectively, these findings indicate that loss of *Dst* in PNS sensory and sympathetic ganglionic neurons results in dysregulated heart rhythms and significant conduction alterations.

**Figure 4:**
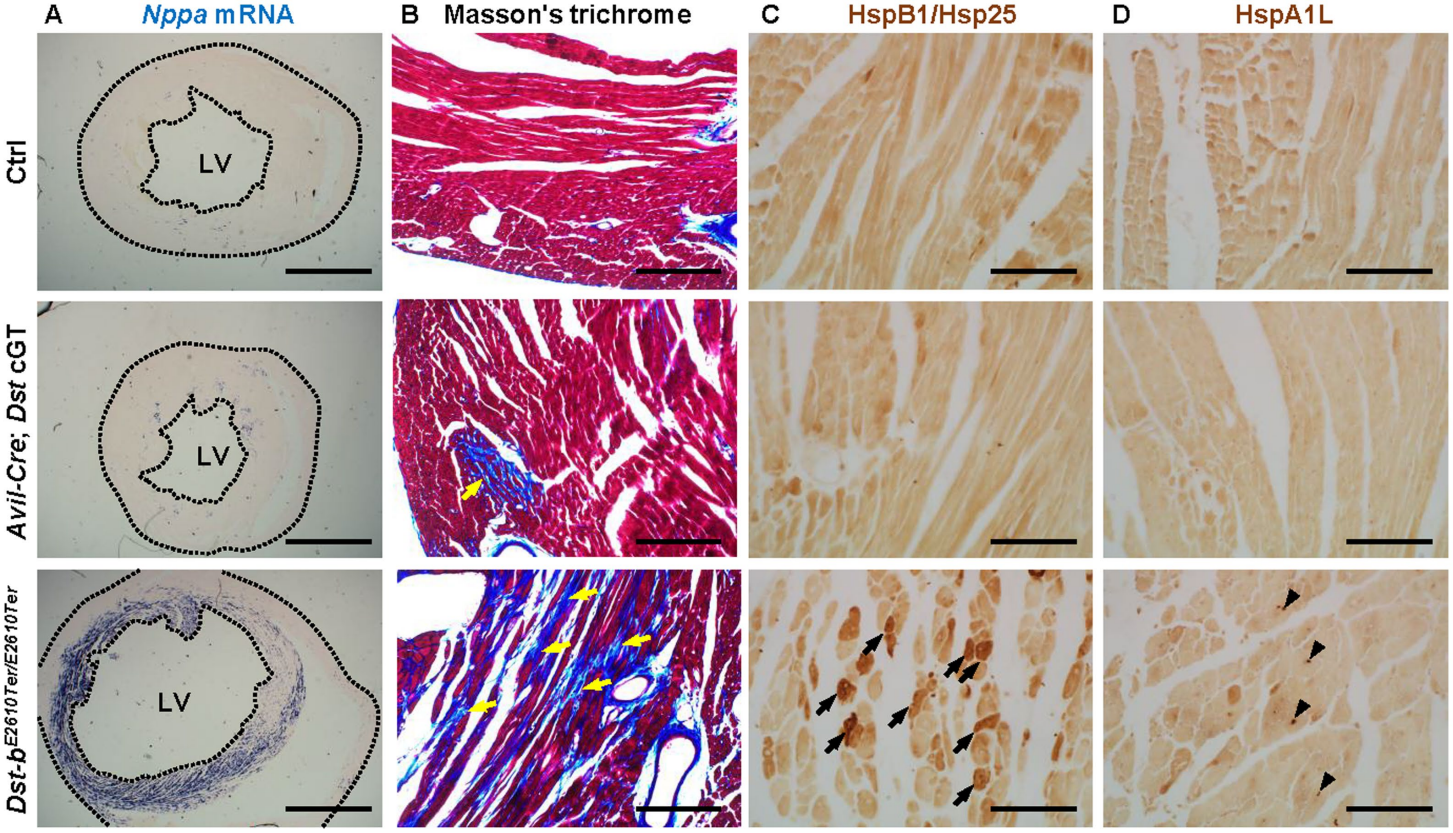
Pathological analyses of cardiac tissues. **(A)** *Nppa in situ* hybridization in the heart at 2 years of age. *Nppa* mRNA expression is upregulated in the left ventricular (LV) myocardium of *Dst-b^E2610Ter/E2610Ter^* mice, but not remarkable in *Avil-Cre*; *Dst* cGT mice or Ctrl mice. Dotted lines indicate tissue boundaries. **(B)** Masson’s trichrome staining showed a sever fibrosis in the myocardium of *Dst-b^E2610Ter/E2610Ter^* mice (yellow arrows). Focal collagen depositions were observed in the myocardium of *Avil-Cre*; *Dst* cGT mice. **(C,D)** Immunohistochemical analyses of heart sections from Ctrl, *Avil-Cre*; *Dst* cGT, and *Dst-b^E2610Ter/E2610Ter^* mice using antibodies against HSPB1/HSP25 **(C)** and HSPA1L **(D)**. HSPB1/HSP25 accumulates in the cytoplasm of cardiomyocytes of *Dst-b^E2610Ter/E2610Ter^* mice (arrows). HSPA1L accumulates in cardiomyocytes as punctate signals (arrowheads). Scale bars, 1,000 μm **(A)**, 100 μm **(B)**, and 50 μm **(C,D)**.

Next, we performed pathological analyses of the heart tissues in *Avil-Cre*; *Dst* cGT mice. We previously reported that loss of the Dst-b isoform from cardiomyocytes leads to cardiomyopathy accompanied by upregulation of the heart failure marker gene natriuretic peptide A (*Nppa*) (Yoshioka et al., 2022). Here, we confirmed upregulation of the *Nppa* transcript in the myocardium of left ventricle from *Dst-b* mutants (*Dst-b*^*E2610Ter*/*E2610Ter*^) at 2 years old (Figure 4A). In contrast, the *Nppa* transcript was sporadically detected in the myocardium of age-matched *Avil-Cre*; *Dst* cGT mice. Cardiac fibrosis was assessed by Masson’s trichrome staining (Figure 4B). A marked increase in the connective tissue was observed in *Dst-b^E2610Ter/E2610Ter^* mice. In contrast, collagen deposition was focal and occurred to a lesser extent in *Avil-Cre*; *Dst* cGT mice. We have also reported that protein aggregate formation is a pathological hallmark of cardiomyopathy in *Dst-b^E2610Ter/E2610Ter^* mice (Yoshioka et al., 2022). Previous RNA sequencing analyses revealed increased expression of chaperone protein-coding genes involved in unfolded protein response (UPR). Here, we performed immunohistochemical analyses of chaperone proteins HspB1/Hsp25 and HspA1L, which are upregulated at the RNA level in *Dst-b^E2610Ter/E2610Ter^* mice (Yoshioka et al., 2022). HspB1/Hsp25 and HspA1L densely aggregated in *Dst-b^E2610Ter/E2610Ter^* cardiomyocytes, displaying distinct staining patterns. HspB1/Hsp25 was diffusely distributed throughout the cytoplasm (Figure 4C), whereas HspA1L-positive aggregates appeared as small punctate signals (Figure 4D). p62 was also observed as dot-like aggregates within the nuclei of *Dst-b^E2610Ter/E2610Ter^* cardiomyocytes (Yoshioka et al., 2022), and immunofluorescent staining confirmed nuclear localization of HspA1L-positive aggregates (Supplementary Figure 5). These findings suggest that alterations in chaperone protein function may contribute to protein aggregate formation in *Dst-b^E2610Ter/E2610Ter^* mice. In the heart tissues of *Avil-Cre*; *Dst* cGT mice, aggregations of HspB1/Hsp25 and HspA1L were scarcely observed. These data suggest that the loss of the Dst-b isoform leads to cardiomyocyte dysfunction and degeneration, whereas the conditional loss of the Dst-a isoform in the PNS circuit does not directly link to severe damage to cardiomyocytes themselves. Therefore, the dysregulation of heart rate in *Avil-Cre*; *Dst* cGT mice is likely caused by neural circuit abnormalities rather than by cardiomyopathy.

### 3.3 Disruption of PNS afferent circuit leads to arrhythmias

**Figure 5:**
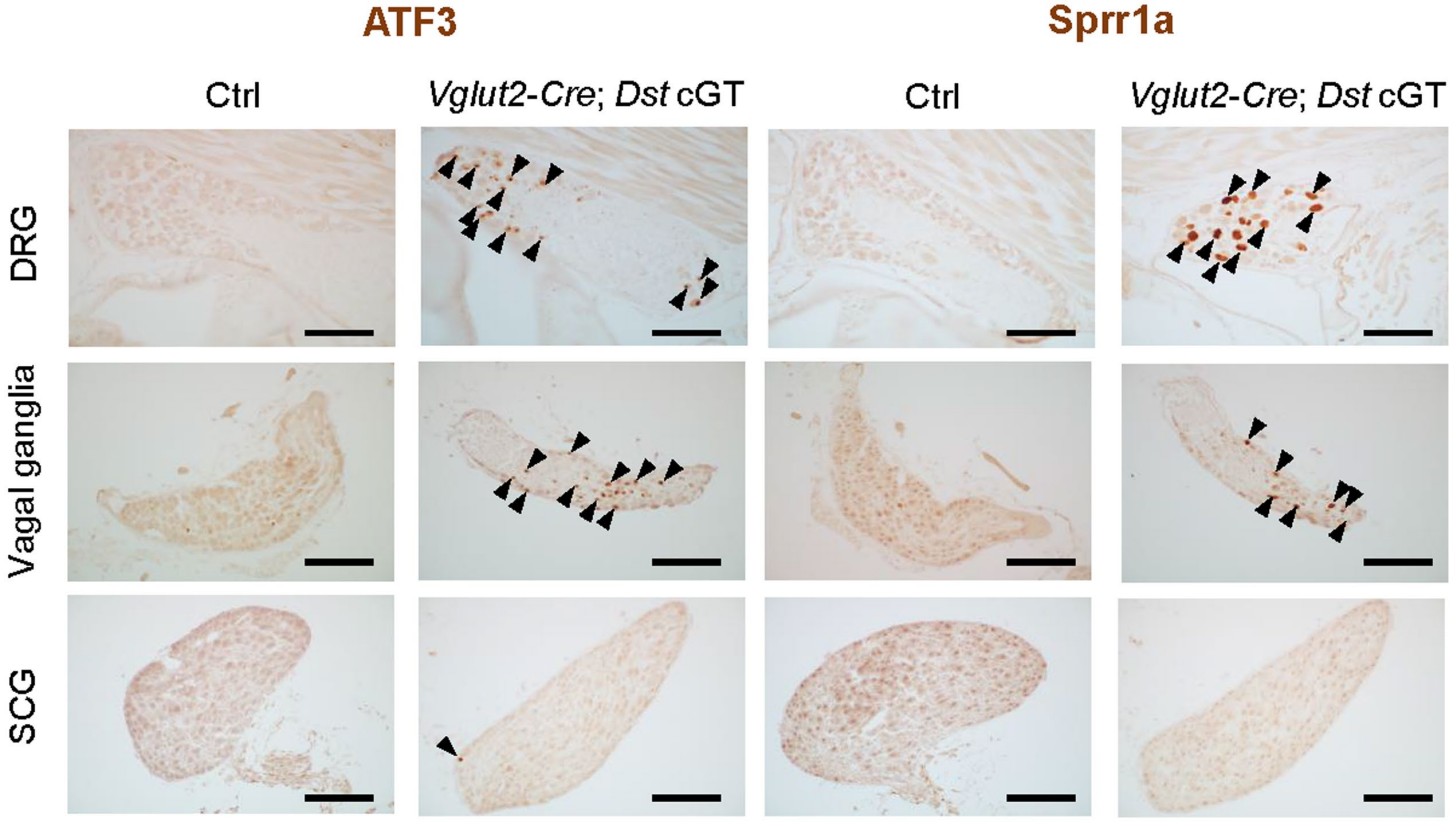
Inactivation of *Dst* expression in sensory neurons causes sensory neurodegeneration. Histological analysis of neurodegenerative changes in the DRG, vagal ganglia, and SCG of *Vglut2-Cre*; *Dst* cGT mice at 4 weeks of age. Expression of the neuronal injury markers ATF3 and Sprr1a is increased in the DRG and vagal ganglia (arrowheads). In contrast, ATF3 and Sprr1a are scarcely expressed in the SCG of *Vglut2-Cre*; *Dst* cGT mice. Scale bars, 100 μm.

Our data suggest that disruption of the PNS neural circuit underlies arrhythmias in *Dst* mutant mice. However, it remains unclear whether abnormalities in the afferent or efferent circuits are involved in the dysregulation of heart rate. To address this issue, we next used *Vglut2-Cre* mice to selectively inactivate the *Dst* expression in sensory neurons, because *Vglut2-Cre*-mediated recombination occurs in sensory neurons but not in sympathetic ganglionic neurons of the PNS (Niu et al., 2020). Although Avil has been accepted to be specifically expressed by PNS neurons (Hunter et al., 2018), vesicular glutamate transporter 2 (Vglut2) is widely expressed by glutamatergic neurons in the central nervous system (CNS) (Kaneko and Fujiyama, 2002). Therefore, we exclusively compare the regions of Cre-mediated recombination in the whole brain and spinal cord of *Avil-Cre*; *Ai14* and *Vglut2-Cre*; *Ai14* after tissue clearing using CUBIC (Supplementary Figure 6). In the brain and spinal cord of *Avil-Cre*; *Ai14*, tdTomato signals were localized in axonal trajectories derived from sensory neurons of the DRG and trigeminal ganglia. In contrast, in *Vglut2-Cre*; *Ai14*, tdTomato-positive cells were distributed throughout the gray matter of the spinal cord, and tdTomato signals were observed throughout the cerebral cortex, where PNS sensory neurons do not directly project. These observations confirmed that *Avil-Cre* is specific to PNS neurons, while *Vglut2-Cre* is active in both CNS and PNS neurons. In the PNS of *Vglut2-Cre*; *Ai14* mice, tdTomato was expressed in sensory neurons of the DRG (Figure 1A) and vagal ganglia (Figure 1B), but not in sympathetic ganglionic neurons of the SCG (Figure 1C). In the SCG, tdTomato was detected only in nerve fibers. These data indicate that Cre-mediated recombination occurs in PNS afferent circuits but not in PNS efferent circuits of *Vglut2-Cre* mice, as expected. Qualitative histological analyses revealed that neural injury markers ATF3 and Sprr1a were induced in sensory neurons of the DRG and vagal ganglia in *Vglut2-Cre*; *Dst* cGT mice, while these markers were scarcely expressed in sympathetic ganglionic neurons of the SCG (Figure 5). These findings indicate that PNS afferent circuits are selectively disrupted in *Vglut2-Cre*; *Dst* cGT mice, whereas PNS efferent circuits remain unaffected.

**Figure 6:**
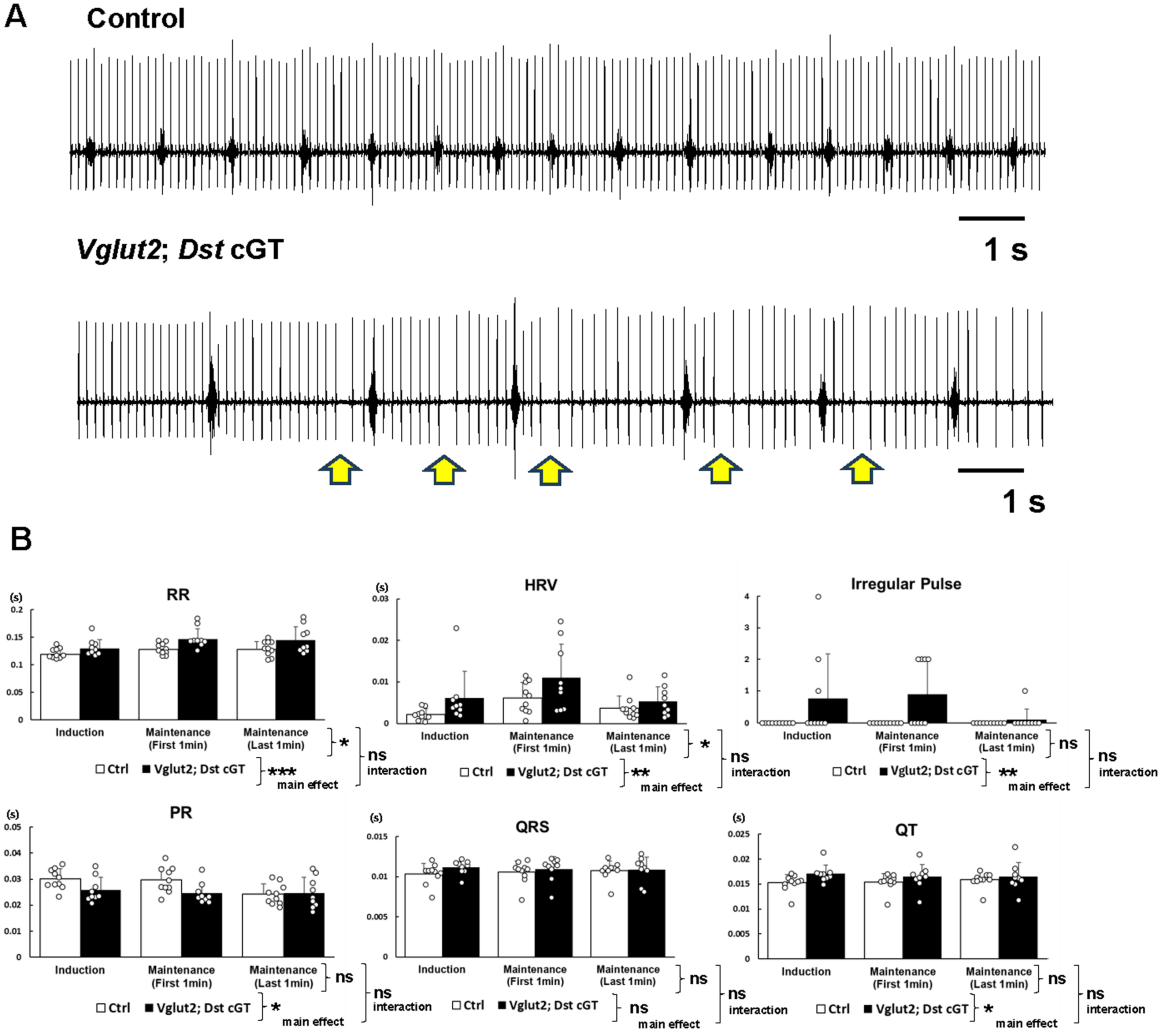
Electrocardiogram recordings from *Vglut2-Cre*; *Dst* cGT mice under anesthesia. **(A)** Representative ECG traces from Ctrl and *Vglut2-Cre*; *Dst* cGT mice under anesthesia. Arrows indicate abnormal skipping of P waves. **(B)** Quantification of mean RR intervals, HRV, frequency of irregular pulse, PR interval, QRS duration, and QT interval (*n* = 10 Ctrl mice; *n* = 9 *Vglut2-Cre*; *Dst* cGT mice, at 11–13 months of age). ECG was quantified in three epochs: (i) 1 min of the induction anesthesia period, (ii) First 1-min of maintenance anesthesia period, and (iii) The final 1 min of the 5-min maintenance period. *, **, and *** denotes statistically significant difference at *p* < 0.05, *p* < 0.01, and *p* < 0.001 and ns means not statistically significant (*p* > 0.05), using two-way ANOVA. Data are presented as mean ± SD.

Finally, we recorded ECG from *Vglut2-Cre*; *Dst* cGT and Ctrl mice at 11–13 months of age. Similar to *Avil-Cre*; *Dst* cGT mice, some *Vglut2-Cre*; *Dst* cGT mice exhibited elevated HRV immediately after the transition to maintenance anesthesia (Figure 6A). Statistically, *Vglut2-Cre*; *Dst* cGT mice exhibited mild but significant increases in HRV (genotype effect, *F* (1, 51) = 7.47, *p* < 0.01; epoch effect, *F* (2, 51) = 4.87, *p* < 0.05; genotype × epoch interaction, *F* (2, 51) = 0.58, *p* > 0.05, two-way ANOVA) and the frequency of irregular pulse (genotype effect, *F* (1, 51) = 10.05, *p* < 0.01; epoch effect, *F* (2, 51) = 1.69, *p* > 0.05; genotype × epoch interaction, *F* (2, 51) = 1.69, *p* > 0.05, two-way ANOVA), as well as a significant shortening of the PR interval compared with Ctrl mice (genotype effect, *F* (1, 51) = 5.63, *p* < 0.05; epoch effect, *F* (2, 51) = 3.05, *p* > 0.05; genotype × epoch interaction, *F* (2, 51) = 2.04, *p* > 0.05, two-way ANOVA) (Figure 6B). QRS complex of *Vglut2-Cre*; *Dst* cGT mice remained unchanged (Figure 6B). Furthermore, unlike the *Avil-Cre* line, *Vglut2-Cre*; *Dst* cGT mice displayed significant alterations in RR (genotype effect, *F* (1, 51) = 13.78, *p* < 0.001; epoch effect, *F* (2, 51) = 3.78, *p* < 0.05; genotype × epoch interaction, *F* (2, 51) = 0.37, *p* > 0.05, two-way ANOVA) and QT intervals (genotype effect, *F* (1, 51) = 4.57, *p* < 0.05; epoch effect, *F* (2, 51) = 0.07, *p* > 0.05; genotype × epoch interaction, *F* (2, 51) = 0.45, *p* > 0.05, two-way ANOVA) (Figure 6B). These data suggest that *Vglut2-Cre*-mediated *Dst* deficiency also leads to rhythmic instability and conduction changes. The specific impact on ventricular depolarization and repolarization differs from that observed in *Avil-Cre*; *Dst* cGT mice. Since isoflurane is known to affect the autonomic nervous system (Kato et al., 1992), we investigated whether cardiac rhythm instability occurs in the conscious state (Supplementary Figure 7). We found that one out of the three *Vglut2-Cre*; *Dst* cGT mice exhibited rhythmic irregularities in the conscious state. These results support the idea that disruption of the PNS afferent circuit, rather than degeneration of sympathetic ganglionic neurons, is a major contributor to dysregulation of the heart rhythm.

## 4 Discussion

The Significance of Disruption of PNS Afferent and Efferent Circuits in Cardiac Dysregulation.

The HSANs comprise complex group of inherited disorders primarily affecting sensory and autonomic nervous systems. Patients with HSANs exhibit multimodal somatosensory impairments, including defects in touch, pain, and proprioception. While somatosensory dysfunction has been extensively investigated, the pathophysiology of visceral dysfunction remains relatively unexplored. Dysregulation of the autonomic nervous system is generally accepted to impair visceral function, leading to a wide range of clinical manifestations such as arrhythmias, orthostatic hypotension, and gastrointestinal motility disorders (Sakae et al., 2001; Thireau et al., 2017). Recent studies using animal models have further elucidated the intricate relationship between autonomic dysfunction and cardiac abnormalities. For example, *Meis1*-deficient mice exhibit abnormal development of sympathetic ganglionic neurons and increased HRV, implicating sympathetic dysfunction in cardiac irregularities (Bouilloux et al., 2016).

The complex mechanisms underlying visceral dysfunction have been investigated using the *Elp1* mutant mouse, a model of familial dysautonomia (FD; HSAN*-*III). Conditional deletion of the *Elp1* gene in sensory and sympathetic ganglionic neurons results in impaired target tissue innervation, supporting the notion that abnormal development of sensory and autonomic neural circuits is a fundamental mechanism of visceral dysfunction (Jackson et al., 2014). The gastrointestinal system is regulated by both extrinsic innervations from the vagus nerve, sympathetic ganglia, and dorsal root ganglia, as well as by the intrinsic enteric nervous system (Sharkey and Mawe, 2023). Conditional deletion of *Elp1* from PNS neurons disrupts normal formation of the enteric nervous system and target innervation of the intestinal mucosa and smooth muscle (Chaverra et al., 2024). Such abnormalities perturb the intestinal epithelial barrier and may underlie gastrointestinal motility disorders in FD patients. Collectively, these animal studies highlight the diverse and complex neural circuit mechanisms contributing to visceral dysfunction in HSANs.

As a model for HSAN-VI, *Dst* mutant mice have also been used to explore the mechanisms underlying gastrointestinal dysfunction (Lynch-Godrei et al., 2020). *Dst* mutants exhibit slowed gastrointestinal motility and thinning of the colonic mucus layer, despite apparently preserved enteric nervous system architecture. Instead, *Dst* mutants show evidence of neurodegeneration in the vagus nerve and vagal ganglia, with little to no degeneration in sympathetic ganglia (Lynch-Godrei et al., 2020), suggesting that impaired parasympathetic control contributes to gastrointestinal dysfunction in HSAN*-*VI. However, the role of visceral afferent circuit abnormalities has remained unclear. In the present study, we demonstrated that PNS neuron-selective ablation of *Dst* gene (*Avil-Cre*; *Dst* cGT) induces upregulations of neural injury markers ATF3 and Sprr1a in sensory neurons of the DRG and vagal ganglia, as well as sympathetic ganglionic neurons of the SCG. Coinciding with previous findings (Lynch-Godrei et al., 2020), neurodegenerative signs were more pronounced in sensory neurons of the vagal ganglia than in sympathetic ganglionic neurons of the SCG. *Avil-Cre*; *Dst* cGT mice exhibited arrhythmias characterized by increased HRV. Notably, milder but similar higher HRV and irregular pulses were observed in *Vglut2-Cre*; *Dst* cGT mice, in which degeneration was restricted to PNS sensory neurons, while sympathetic ganglionic neurons remained intact. It suggests that the increased HRV is primarily driven by the degeneration of PNS sensory neurons. In contrast, the differences in ventricular depolarization and repolarization between the two models may result from distinct recombination in other regions, including the CNS, rather than sensory circuits alone. Furthermore, while *Vglut2-Cre* does not induce direct recombination within the autonomic neurons (Chang et al., 2015), it may indirectly modulate autonomic nervous system since Vglut2-positive interneurons are known to regulate structural and functional plasticity of sympathetic nervous systems after spinal cord injury (Noble et al., 2022). These conditional gene-targeting strategies support the concept that degeneration of PNS sensory neurons leads to the disturbance of visceral afferent circuits and subsequent dysregulation of heart rhythms via disruption of interactions between visceral afferent and efferent circuits. However, it should be noted that the relationship observed between these specific regions of neurodegeneration and the onset of arrhythmia remains correlative, and there are technical limitations in establishing a definitive causative link within the current framework. In recent years, the use of viral vectors and optogenetics has allowed precise mapping of the peripheral neural circuits involved in heart rate control (Rajendran et al., 2019). In future studies, more localized and temporal manipulation of *Dst* expression will elucidate the direct causal relationship between sensory circuit disruption and arrhythmogenesis. Furthermore, it is well-established that isoflurane anesthesia disrupts autonomic nervous system balance (Kato et al., 1992). While the occurrence of arrhythmias in the awake state indicates that autonomic dysfunction and rhythm disturbances do not always manifest concurrently, the significantly increased frequency of these events under isoflurane anesthesia suggests that *Dst* mutant mice possess a heightened vulnerability to autonomic stressors. This increased susceptibility likely reflects impaired homeostatic resilience, highlighting the critical role of sensory circuits in maintaining visceral homeostasis.

### 4.1 PNS afferent control of heart rate via multiple feedback circuits

Visceral sensation is generally attributed to sensory neurons in vagal ganglia (Waise et al., 2018; Ichiki et al., 2022; Prescott and Liberles, 2022). Recent optogenetic and viral tracing studies have demonstrated that afferent signals conveyed via the vagus nerve enhance parasympathetic tone and suppress sympathetic activity to the heart through the CNS pathways (Rajendran et al., 2019). In contrast, other studies have identified tropomyosin receptor kinase C (TrkC)-positive sensory neurons in the DRG that regulate blood pressure and heart rate via sympathetic nervous system pathways (Morelli et al., 2021). Together, these findings indicate that PNS sensory neurons control cardiovascular function through multiple feedback circuits involving the autonomic nervous system. In the present study, sensory neuron-selective loss of the *Dst* leads to severe degeneration of sensory neurons in both the vagal ganglia and DRG, suggesting a simultaneous disruption of the visceral feedback circuit through these distinct routes. DRG neurons are derived exclusively from the neural crest, whereas vagal ganglia arise from a combination of neural crest cells and ectodermal placodes. This dual developmental origin may contribute to the pathological heterogeneity observed in visceral sensory deficits among HSANs. Elucidating how these developmental differences influence the differential vulnerability of sensory neuron populations will be an important direction for future research.

### 4.2 The mechanisms of pathogenesis of visceral dysfunctions in HSAN

The pathophysiology of visceral dysfunction in HSANs, including cardiovascular reflex abnormalities, gastrointestinal dysfunction, and respiratory failure, is multifactorial and involves complex interactions among multiple organs. The *Dst* gene encodes tissue-selective isoforms DST-a, DST-b, and DST-e expressed predominantly in neural, muscular, and cutaneous tissues, respectively (Künzli et al., 2016). These Dst isoforms play critical roles in maintaining tissue homeostasis (Yoshioka, 2024). Our initial analysis of *Dst-b* mutant mice predicted the presence of muscle pathology (Yoshioka et al., 2022), leading to the identification of patients with congenital myopathy and cardiomyopathy associated with *DST-b* mutations (Jacob et al., 2025). The *Dst-b* mutation induces protein aggregate formation accompanied by accumulation of chaperone proteins associated with the UPR. Consequently, *Dst-b* mutant mice exhibit arrhythmias, including QT prolongation and premature contraction with protein aggregate cardiomyopathy (Yoshioka et al., 2022). In contrast, PNS neuron-selective *Dst* cGT mice (*Avil-Cre*; *Dst* cGT and *Vglut2-Cre*; *Dst* cGT) displayed sinus arrhythmias without the formation of protein aggregates. Although *Avil-Cre*; *Dst* cGT mice exhibited less severe cardiomyocyte damage compared to *Dst-b* mutant mice, the presence of mild fibrosis suggests that the PNS may contribute to the maintenance of cardiomyocytes. Therefore, the mechanisms by which peripheral nerve dysfunction mediates myocardial damage remain an important topic for future research. These findings suggest that *DST* mutations can cause arrhythmia through multiple mechanisms, including intrinsic cardiomyocyte dysfunction and the disruption of neural circuits regulating cardiac functions. Respiratory failure has also been also reported as a cause of juvenile death in patients with severe form of HSAN*-*VI (Edvardson et al., 2012; Ahmad et al., 2025). Notably, vagal ganglia contain sensory neurons that project to both the cardiovascular and respiratory systems, potentially mediating coordination between respiration and heart rate (Devarajan et al., 2022). Given the established association between cardiac arrhythmias and respiratory control, this interaction should be considered in the pathophysiological mechanisms underlying arrhythmias in HSANs. Combining these findings, it is necessary to elucidate visceral dysfunction in HSANs from a comprehensive perspective that integrates the roles of causative genes, neural circuits, and multi-organ interactions.

## Data Availability

The raw data supporting the conclusions of this article will be made available by the authors, without undue reservation.
