# Supplementary material for: Disruption of afferent neural circuits leads to arrhythmia in the animal model of hereditary sensory and autonomic neuropathy 6
Source: Front Neural Circuits. 2026 Apr 8;20:1777115. doi: 10.3389/fncir.2026.1777115 (PMC13099889; doi:10.3389/fncir.2026.1777115)
Supplement: Supplementary file 1 [file Data_Sheet_1.pdf]

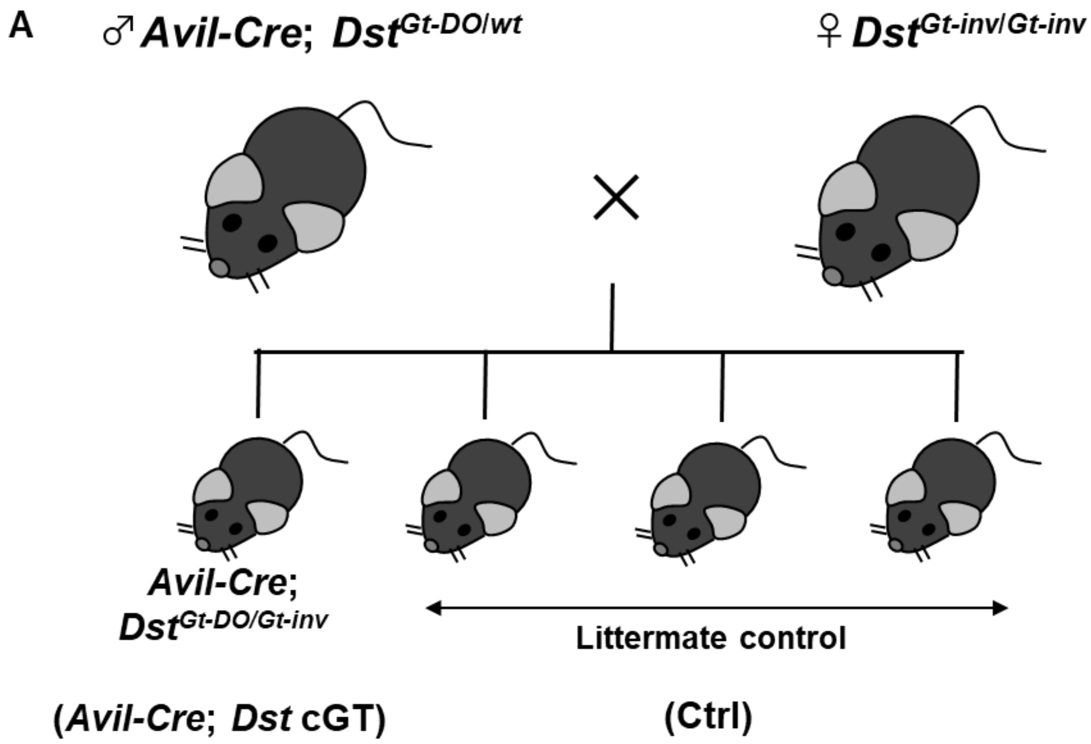

Supplemental Figure S1. Mating scheme to generate PNS neuron-selective *Dst* cGT mice.

(A) Breeding scheme to generate *Avil-Cre*; *Dst* cGT mice is shown.

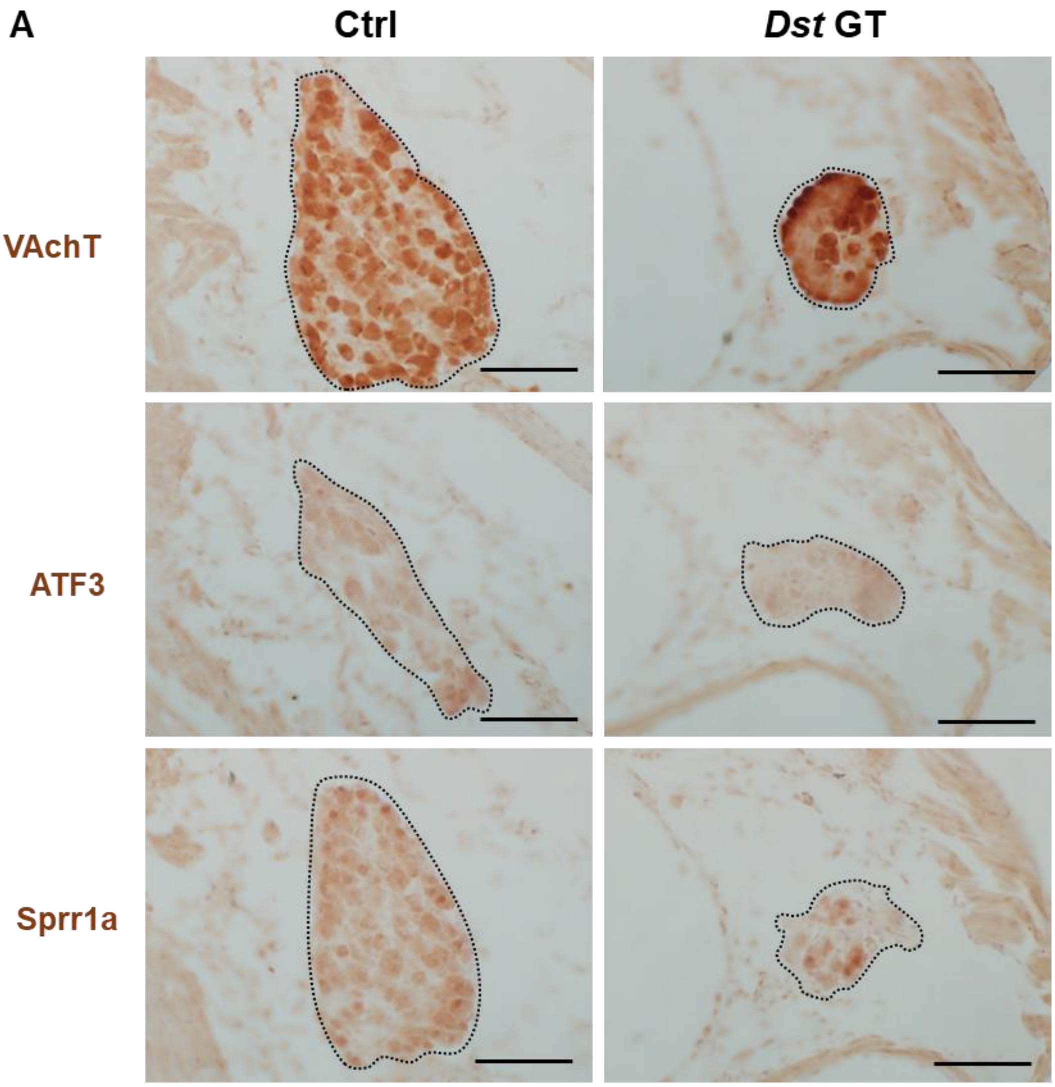

Supplemental Figure S2. Histological analyses in cardiac ganglia of *Dst* GT mice.

(A) Histological analysis in cardiac ganglia of *Dst* GT mice. Dotted lines indicate boundaries of the cardiac ganglia. The parasympathetic ganglionic neuron marker VachT is expressed by cardiac ganglia. Expression of the neuronal injury markers ATF3 and Sprr1a is scarcely expressed in the cardiac ganglia of *Dst* GT mice likely to Ctrl mice. Scale bars, 50 µm.

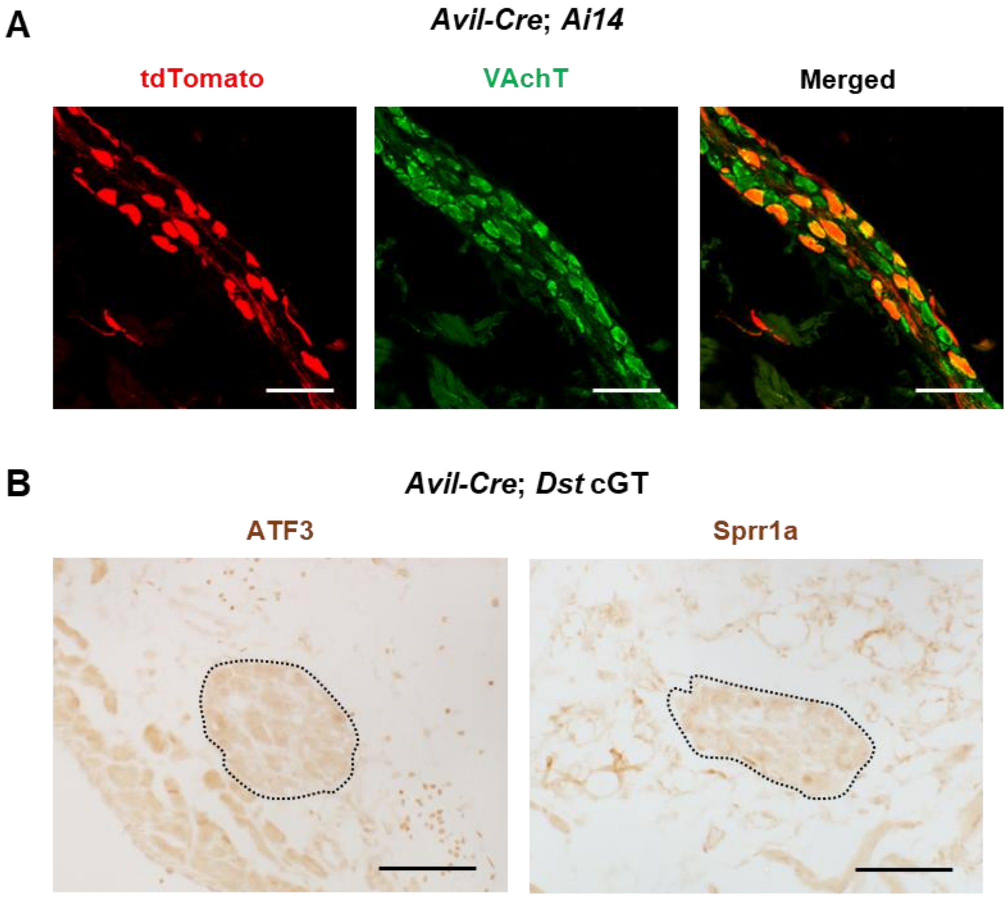

Supplemental Figure S3. Histological analyses in cardiac ganglia of *Avil-Cre*; *Ai14* and *Avil-Cre*; *Dst* cGT mice.

(A) The expression of tdTomato was shown in the cardiac ganglia of *Avil-Cre*; *Ai14* mice. tdTomato is expressed by parasympathetic ganglionic neurons positive for VachT. (B) Histological analysis of neurodegenerative changes in the cardiac ganglia of *Avil-Cre*; *Dst* cGT mice at 4 weeks of age. Dotted lines indicate boundaries of the cardiac ganglia. Expression of the neuronal injury markers ATF3 and Sprr1a is scarcely expressed in the cardiac ganglia of *Avil-Cre*; *Dst* cGT mice. Scale bars, 50 µm (A, B).

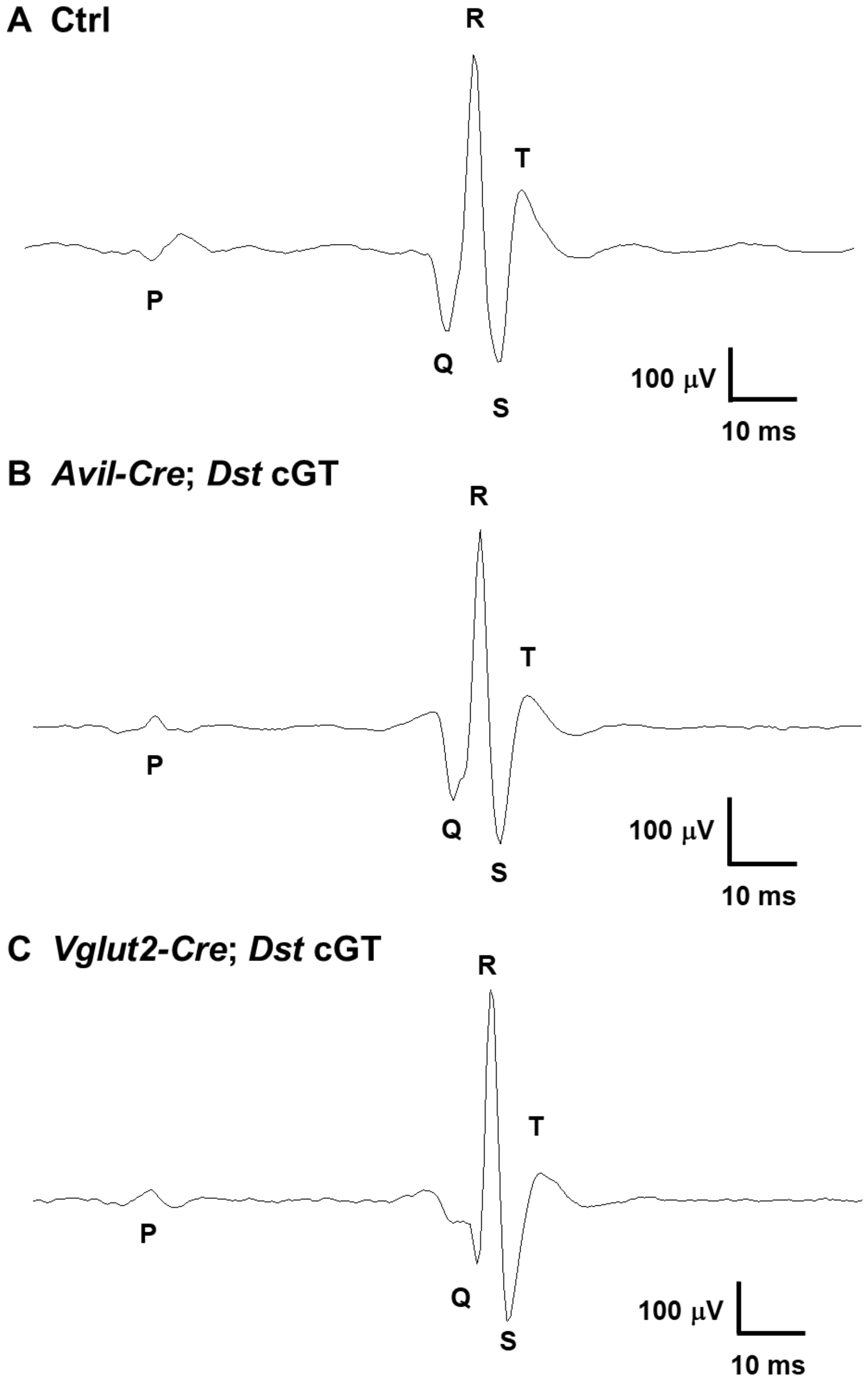

Supplemental Figure S4. Representative trace of P-QRS-T complex.

(A-C) A representative ECG image of P-QRS-T complex recorded from Ctrl (A), *Avil-Cre*; *Dst* cGT (B) and *Vglut2-Cre*; *Dst* cGT (C) mice are shown.

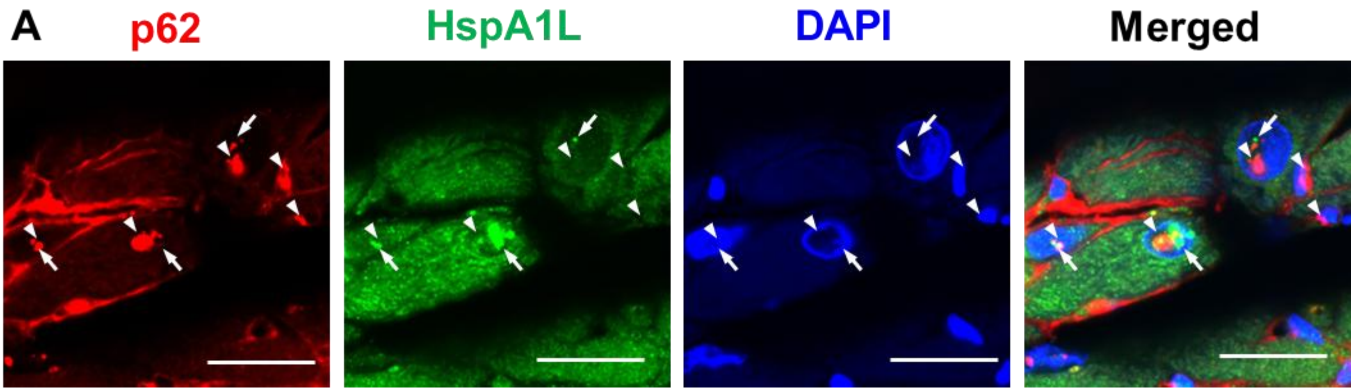

Supplemental Figure S5. Histological features of intranuclear structures in *Dst-b*$^{E2610Ter/E2610Ter}$ cardiomyocytes. (A) Immunofluorescence images of protein aggregates in cardiomyocytes from *Dst-b*$^{E2610Ter/E2610Ter}$ mice. p62- and HspA1L-positive structures are located within the nucleus. Arrowheads and arrows indicate depositions of p62 and HspA1L, respectively. p62 and HspA1L are detected in distinct intranuclear structures. Scale bars: 10 μm.

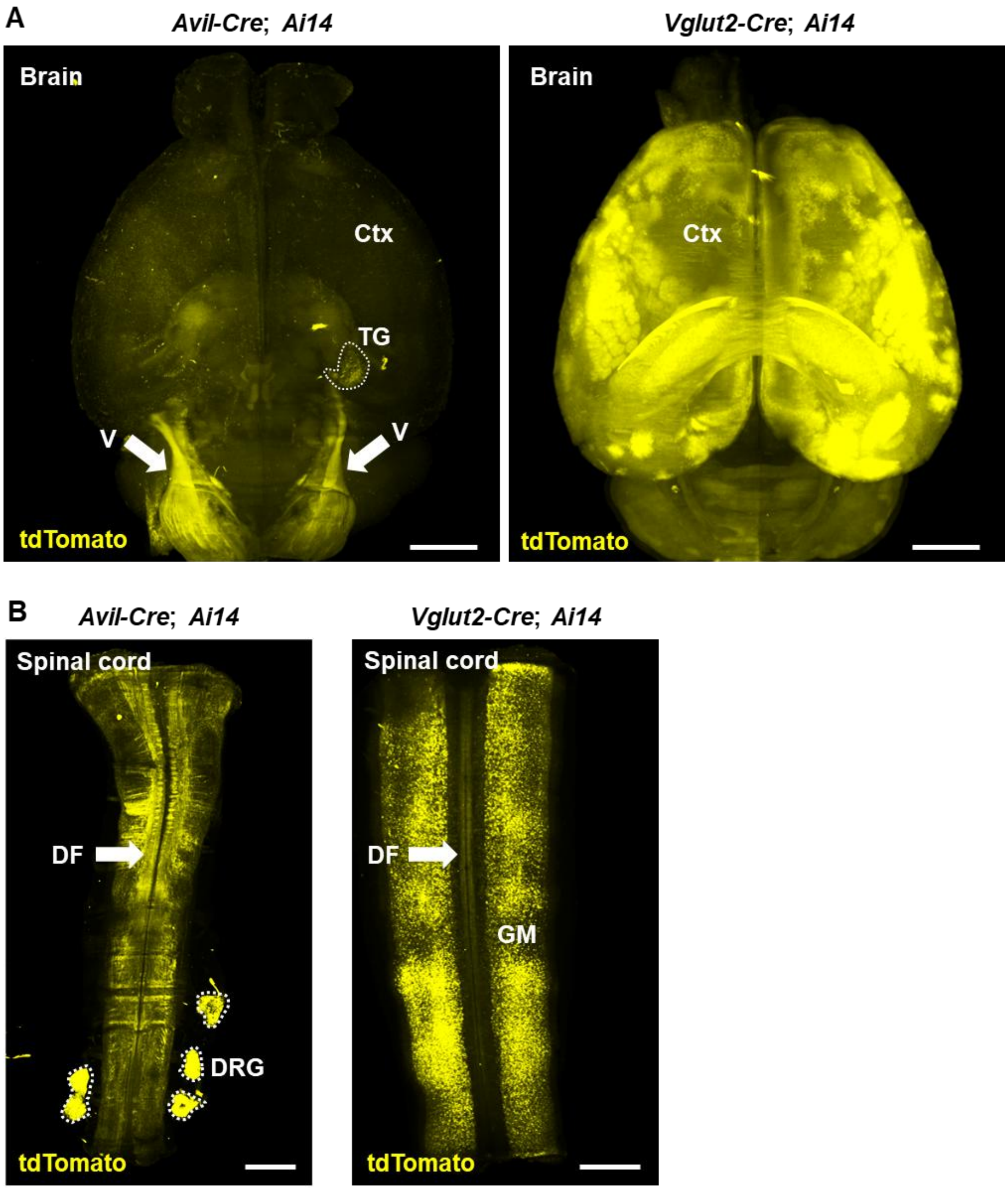

Supplemental Figure S6. 3D imaging of the nervous system in *Avil-Cre*; *Ai14* and *Vglut2-Cre*; *Ai14* mice. (A-B) Reconstructed transparent images of whole brain and spinal cord from *Avil-Cre*; *Ai14* and *Vglut2-Cre*; *Ai14* mice. (A) In the brain of an *Avil-Cre*; *Ai14* mouse, tdTomato is localized specifically to the trigeminal ganglia (TG) and their axons in trigeminal nerve (V, arrows). In contrast, *Vglut2-Cre*; *Ai14* mice exhibit widespread tdTomato fluorescence in the cerebral cortex (Ctx). Since the Ctx receives no direct projections from primary sensory neurons, these signals are attributable to expression by CNS neurons. (B) In an *Avil-Cre*; *Ai14* mouse, tdTomato labeling is localized to the DRG and their ascending axons within the spinal dorsal funiculus (DF, arrows). However, in an *Vglut2-Cre*; *Ai14* mouse, tdTomato is broadly expressed in neurons across the gray matter (GM), as well as in axons within the DF. Scale bars: 1 mm.

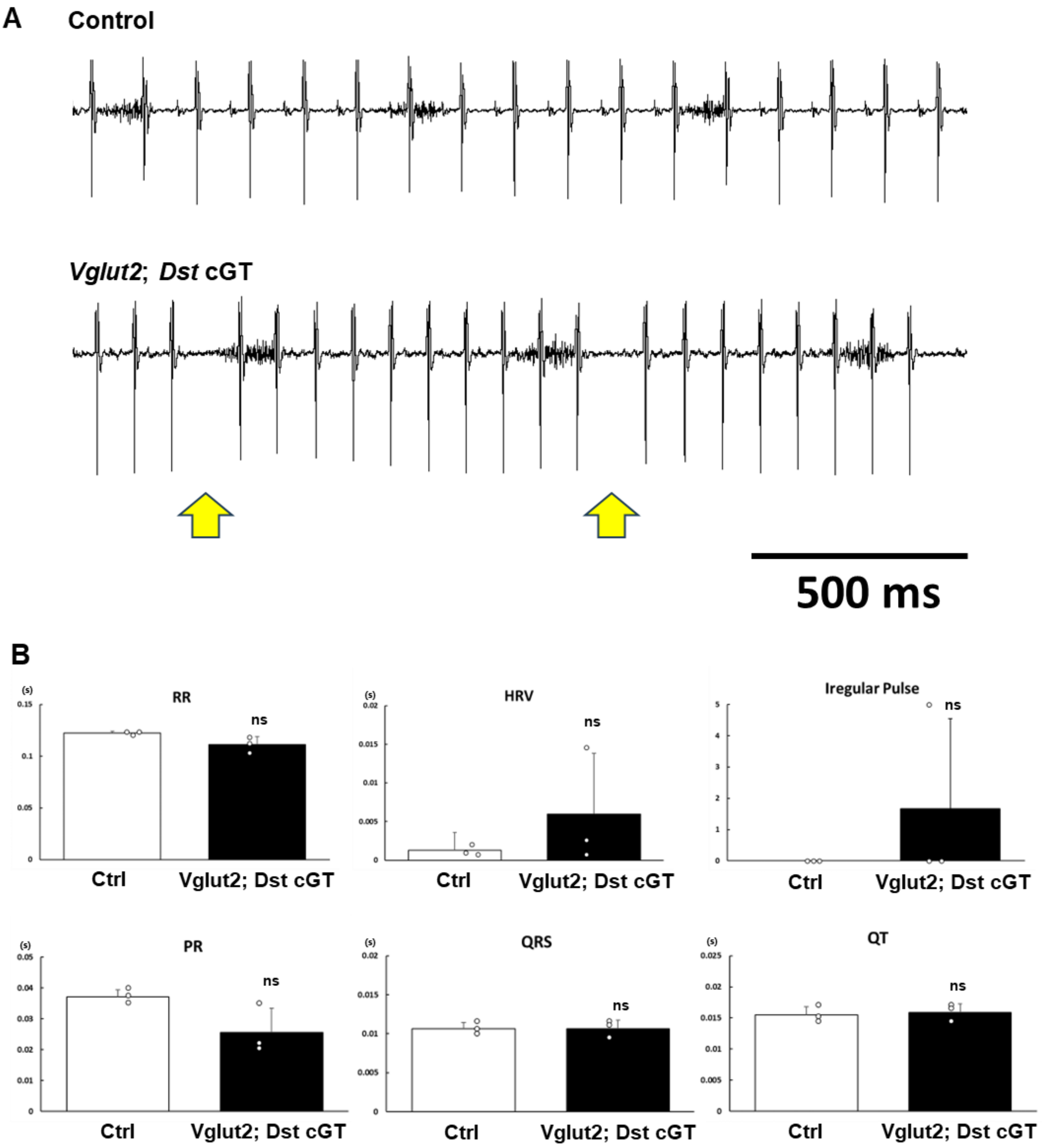

Supplemental Figure S7. Electrocardiogram recordings from awake *Vglut2-Cre*; *Dst* cGT mice

(A) Representative ECG traces from awake Ctrl and *Vglut2-Cre*; *Dst* cGT mice. Arrows point to the abnormal skipping of P waves. (B) Quantification of mean RR intervals, HRV, frequency of irregular pulse, PR interval, QRS duration, and QT interval (n = 3 Ctrl mice; n = 3 *Vglut2-Cre*; *Dst* cGT mice, at 15–17 months of age). ns means not statistically significant ($p > 0.05$), using *t*-test. Data are presented as mean ± SD.
